# Glycosylation of Ca_V_3.2 Channels Contributes to the Hyperalgesia in Peripheral Neuropathy of Type 1 Diabetes

**DOI:** 10.3389/fncel.2020.605312

**Published:** 2020-12-15

**Authors:** Sonja Lj. Joksimovic, J. Grayson Evans, William E. McIntire, Peihan Orestes, Paula Q. Barrett, Vesna Jevtovic-Todorovic, Slobodan M. Todorovic

**Affiliations:** ^1^Department of Anesthesiology, University of Colorado Denver, Aurora, CO, United States; ^2^Undergraduate School of Arts and Sciences, University of Virginia, Charlottesville, VA, United States; ^3^Department of Molecular Physiology and Biological Physics, University of Virginia Health System, Charlottesville, VA, United States; ^4^Department of Anesthesiology, University of Virginia Health System, Charlottesville, VA, United States; ^5^Department of Pharmacology, University of Virginia, Charlottesville, VA, United States; ^6^Neuroscience Graduate Program and Graduate Program in Pharmacology, University of Colorado Denver, Aurora, CO, United States

**Keywords:** dorsal root ganglion, T-type calcium channel, neuropathic pain, sensitization, excitability, low-voltage-activated

## Abstract

Our previous studies implicated glycosylation of the Ca_V_3.2 isoform of T-type Ca^2+^ channels (T-channels) in the development of Type 2 painful peripheral diabetic neuropathy (PDN). Here we investigated biophysical mechanisms underlying the modulation of recombinant Ca_V_3.2 channel by de-glycosylation enzymes such as neuraminidase (NEU) and PNGase-F (PNG), as well as their behavioral and biochemical effects in painful PDN Type 1. In our *in vitro* study we used whole-cell recordings of current-voltage relationships to confirm that Ca_V_3.2 current densities were decreased ~2-fold after de-glycosylation. Furthermore, de-glycosylation induced a significant depolarizing shift in the steady-state relationships for activation and inactivation while producing little effects on the kinetics of current deactivation and recovery from inactivation. PDN was induced *in vivo* by injections of streptozotocin (STZ) in adult female C57Bl/6j wild type (WT) mice, adult female Sprague Dawley rats and Ca_V_3.2 knock-out (KO mice). Either NEU or vehicle (saline) were locally injected into the right hind paws or intrathecally. We found that injections of NEU, but not vehicle, completely reversed thermal and mechanical hyperalgesia in diabetic WT rats and mice. In contrast, NEU did not alter baseline thermal and mechanical sensitivity in the Ca_V_3.2 KO mice which also failed to develop painful PDN. Finally, we used biochemical methods with gel-shift analysis to directly demonstrate that N-terminal fragments of native Ca_V_3.2 channels in the dorsal root ganglia (DRG) are glycosylated in both healthy and diabetic animals. Our results demonstrate that in sensory neurons glycosylation-induced alterations in Ca_V_3.2 channels *in vivo* directly enhance diabetic hyperalgesia, and that glycosylation inhibitors can be used to ameliorate painful symptoms in Type 1 diabetes. We expect that our studies may lead to a better understanding of the molecular mechanisms underlying painful PDN in an effort to facilitate the discovery of novel treatments for this intractable disease.

## Introduction

A recent report by the World Health Organization estimated that over 422 million people worldwide suffered from diabetes in 2014 (https://www.who.int/news-room/fact-sheets/detail/diabetes). Despite significant advances in therapy, people with diabetes still struggle to maintain normoglycemia, placing them at increased risk for the development of diabetic complications, including peripheral diabetic neuropathy (PDN). This condition is characterized by peripheral nerve damage caused by chronic hyperglycemia. Most PDN patients will develop abnormal sensation in their extremities either in the form of painful PDN (typically early on) or painless (insensate) PDN in the late stages of diabetes (Obrosova, 2009). Both intractable pain and loss of pain sensation have significant detrimental effects on one's quality of life. Further, none of the currently available treatments can completely reverse these symptoms. The prevalence of chronic pain is estimated to affect 10–20% of patients with diabetes and 40–50% of those patients will experience PDN (Veves et al., 2008). A substantial real-life data from clinical experience indicates that calcium channel α2δ ligands, serotonin and noradrenaline reuptake inhibitors (SNRIs) and tricyclic antidepressants (TCAs) are the most efficient pharmacological treatments of PDN, however the lack of comparative efficacy studies, cost and occurrence of serious adverse events are hindering the use of these conventional therapies (Feldman et al., 2019). Hence, new mechanism-based therapies for PDN are needed in order to improve treatment of this debilitating condition.

We have previously demonstrated that the Ca_V_3.2 isoform of T-type calcium channels (T-channels) in peripheral sensory neurons contributes to the hyperexcitability of sensory neurons in both physiological and pathological conditions, resulting in two frequent symptoms of chronic neuropathic pain: hyperalgesia and allodynia (Nelson and Todorovic, 2006; Todorovic and Jevtovic-Todorovic, 2011). Specifically, our previous animal studies established that in both type 1 and type 2 diabetes the Ca_V_3.2 isoform of T-channel family plays a key role in sensitization of pain responses in PDN by enhancing the excitability of peripheral nociceptors of dorsal root ganglia (DRG) (Jagodic et al., 2007; Latham et al., 2009; Messinger et al., 2009; Choe et al., 2011; Orestes et al., 2013). In addition, we found that in a leptin-deficient (ob/ob) mouse model of type 2 PDN de-glycosylation treatment with neuraminidase (NEU) inhibited native T-currents in sensory neurons *in vitro* and reversed hyperalgesia *in vivo* (Orestes et al., 2013).

Here, we sought to obtain a more detailed understanding of how post-translational glycosylation influences both the biophysical properties of the Ca_V_3.2 isoform and the subsequent development of hyperalgesia and allodynia in type 1 PDN model that was induced by injections of streptozotocin (STZ) in rats or mice. In order to investigate the effects of NEU and PNGase-F (PNG) induced de-glycosylation of Ca_V_3.2 channels on Ca_V_3.2 current kinetics, we recorded calcium currents in human embryonic kidney cells 293 (HEK293) expressing Ca_V_3.2 channels that were grown and maintained in hyperglycemic conditions before and after treatment with de-glycosylating enzymes. We investigated *in vivo* effects of NEU injection on nociceptive thresholds of STZ-treated rats or mice. Finally, we used biochemical methods with gel-shift analysis to directly demonstrate that the N-terminal fragments of native Ca_V_3.2 channels in the rat dorsal root ganglia (DRGs) are glycosylated in both physiological and pathological conditions.

## Materials and Methods

### Animals

All methods for use of rats and mice are described in details in our recent publication (Joksimovic et al., 2020) and are only briefly described here. Experimental protocols were approved by the Animal Care and Use Committee of the University of Colorado Anschutz Medical Campus, as well as University of Virginia. All animals were housed 2 per cage, on a 12 h light-dark cycle with food and water *ad libitum*. Young adolescent female Sprague-Dawley (Envigo, Indianapolis, IN, USA) rats (8–12 weeks of age) were used for the biochemical and *in vivo* studies. Adult female C57BL/6j and Ca_V_3.2 (CACNA1H) knock out (KO) mice of the same source (The Jackson Laboratory) were used for the behavioral experiments. There is growing scientific evidence suggesting sex differences in pain perception, with females being more susceptible to both acute and chronic pain conditions than males (Bartley and Fillingim, 2013). Surprisingly, the vast majority of animal pain studies are performed in males (Mogil and Chanda, 2005). Hence, in order to better understand the underlying mechanisms of pain perception associated with Type 1 PDN, we used only female rats and mice in our experiments.

On each experimental day, animals were randomly assigned to treatment groups with the experimenter blinded to drug treatment. All efforts were made to reduce animal suffering and the number of animals used. In *in vivo* experiments, all groups consisted of 4 or more animals. Whenever possible, studies were designed to generate groups of equal size, using randomization and blinded analysis (Joksimovic et al., 2018, 2020).

### Induction of Diabetes Type I in Rodents

In rodents hyperglycemia and diabetes type I were induced with one single high dose injection of streptozotocin as previously described (Furman, 2015). Streptozotocin solution was prepared immediately prior to injection by dissolving it in sodium citrate buffer (pH 4.5) to a final concentration of 20 mg/ml. Upon preparation, STZ solution was injected as a single intraperitoneal (i.p.) injection (65 mg/kg dose for rats and 200 mg/kg dose for mice) in animals that previously underwent fasting (4 h for mice and 6–8 h for rats). Upon injection, animals were transferred to their cages and fed with normal food and 10% sucrose water. Animals were closely monitored every 2 h for total of 12 h for marked hypoactivity, unresponsiveness or convulsions. On a second day post-injection, 10% sucrose water was replaced with regular water. To confirm the development of diabetes, fasting blood glucose levels were taken daily following STZ injection.

### Thermal Nociception Testing

All methods for *in vivo* assessment of thermal nociception are described in details in our recent publication (Joksimovic et al., 2020) and are only briefly described here. For assessment of thermal (heat) nociception threshold, an apparatus based upon Hargreaves method was used (custom created at UCSD University Anesthesia Research and Development Group, La Jolla, CA). In brief, after 15 min of acclimation, a radiant heat source is positioned directly underneath a plantar surface of the hind paws to deliver a thermal stimulus. When the animal withdraws the paw, an automatic timer shuts off measuring the animal's paw withdrawal latency (PWL). Each paw was tested three times and the average value of PWLs was used in further analysis. To prevent thermal injury, the light beam is automatically discontinued at 20 s if the rat fails to withdraw its paw.

### Mechanical Sensitivity

All methods for *in vivo* assessment of mechanical hyperalgesia are described in details in our recent publication (Joksimovic et al., 2020) and are only briefly described here. To determine mechanical sensitivity in rats, we used the electronic Von Frey apparatus (Ugo Basile, Varese, Italy). The apparatus utilizes a single rigid filament that exerts pressure to the plantar surface of the paw in a range from 0 to 50 grams. Animals were placed in plastic enclosures on a wire mesh stand to habituate for 15 min. After habituation, a probe was applied to the plantar surface of the paw through the mesh floor of the stand, and constant force was applied to the mid-plantar area of the paw. As soon as the exerted pressure of the punctate stimulus reaches the maximum force that the animal can endure, immediate brisk paw withdrawal is noticeable, and the force in grams is displayed on the apparatus representing a threshold for paw withdrawal response (PWR). Each paw was tested three times and the average value of threshold PWRs was used in further analysis. Any other voluntary movement of the animal was not considered as a response.

### Biochemical Methods

Several dorsal root ganglia (DRGs) were harvested from either wild type or diabetic rats, flash frozen in liquid nitrogen and stored at −80°C. Tissue was homogenized by 11 repeated freeze thaw cycles in liquid nitrogen and repeated tissue homogenization with pestle; samples were sonicated with 5–10 pulses in sonicator with 20% amplitude; further homogenization was achieved by adding RIPA buffer (Thermo Scientific) and protease inhibitors, and passing homogenate repeatedly through a 21 g needle; all purification steps were performed at 4°C unless otherwise noted. Fragments of the Cav3.2 channel containing the nona-histidine motif (amino acids 521-529) were captured using Ni-NTA (Qiagen) beads added directly to the RIPA cell lysate following syringe homogenization. After a 2-h incubation, the Ni-NTA beads were washed with three 500 μl volumes of Ni-NTA wash buffer (20 mM HEPES, pH 7.5, 150 mM NaCl, 0.1% *n-*dodecyl-D-maltoside, 0.02% cholesteryl hemisuccinate and protease inhibitors). Proteins that remained bound to the Ni-NTA beads were eluted with Ni-NTA wash buffer containing 200 mM imidazole. De-glycosylation was performed by incubation of samples with 500 units of PNGaseF (New England BioLabs) for 45 min at room temperature.

Samples were separated by SDS-PAGE using either 4–20% gradient Mini-PROTEAN TGX gels (BioRad), or 14 × 16 cm 6% gels. Proteins were visualized by staining with Simply Blue Safestain (Novex), or transferred to nitrocellulose using a Mini Trans-Blot Electrophoretic Transfer cell (BioRad) at 100 volts for 1 h. After transfer, membranes were blocked with a solution of TBST containing 5% milk, and subsequently incubated with the polyclonal antibody LS-C153507 (LifeSpan BioSciences), which was raised against a synthetic peptide derived from the N-terminal domain of human Ca_V_3.2. Unbound antibody was removed with two 5 min TBST washes, and blots were incubated with fluorescent IRDye 800CW donkey anti-rabbit secondary antibody. Fluorescence was detected using an Odyssey imager (Li-Cor).

### Cell Culture

Cultured human embryonic kidney (HEK) 293 cells with stable expression of Ca_V_3.2 channels were grown in Dulbecco's modified Eagle's medium containing 10% FBS (Orestes et al., 2013). Stable cell lines expressing the epitope-tagged Ca_V_3.2 channel were selected using the above media containing 500 μg/mL G418. For electrophysiology recordings cells were typically used 1–3 days after plating.

### Electrophysiology

All methods for *in vitro* recordings of calcium currents are described in details in our recent publication (Joksimovic et al., 2020) and are only briefly described here. The external solution for voltage-clamp experiments measuring T-currents in HEK293 cells contained 152 mM tetraethylammonium (TEA)-Cl, 2 mM CaCl2, and 10 mM HEPES, with TEA-OH, which was used for adjustment of pH to 7.4. In some experiments, 2 mM CaCl2 in external solution was replaced with 10 mM BaCl2. The internal solution contained 135 mM tetramethyilammonium (TMA)-OH, 40 mM HEPES, 10 mM EGTA, and 2 mM MgCl2, adjusted to pH 7.2 with hydrogen fluoride. Series resistance (Rs) and membrane capacitance (Cm) were recorded directly from the amplifier after electronic subtraction of the capacitive transients. Current-voltage (I-V) curves were generated by voltage steps from holding potentials (Vh) of −90 mV to test potentials (Vt) from −80 to −30 mV in incremental steps of 5 mV. The voltage dependence of activation was determined with a single Boltzmann distribution:

(1) $$\begin{array}{l} \text{Activation},\text{ I}\left(\text{V}\right)={\text{I}}_{\text{max}}/\left(1+\text{exp}\left[-\left(\text{V}-{\text{V}}_{50}\right)/k\right]\right) \end{array}$$

The voltage dependence of inactivation was determined with single Boltzmann distribution:

(2) $$\begin{array}{l} \text{inactivation},\text{I}\left(\text{V}\right)={\text{I}}_{\text{max}}/\left(1+\text{exp}\left[\left(\text{V}-{\text{V}}_{50}\right)/k\right]\right) \end{array}$$

Time course (tau) of deactivation and macroscopic current inactivation was obtained by fitting a single term exponential function. Time course of recovery from inactivation was obtained by fitting a double term exponential function yielding tau (τ) 1 and tau (τ) 2.

Interestingly, we determined that the glucose level in Invitrogen's DMEM routinely used to grow and maintain HEK923 cells is 315 mg/dL (17.5 mmol/L) glucose, a value similar to blood glucose levels in diabetic mice (Orestes et al., 2013). To study effects of de-glycosylation on the recombinant Ca_V_3.2 current kinetics, we exposed recombinant Ca_V_3.2 channels to neuraminidase (NEU; at 1.5 U/ml for 1–3 h at 37°C), an enzyme that de-glycosylates proteins by removing sialic acid residues, or PNGase-F (PNG; at 20U/cc for 12 h at 37° C) an enzyme that selectively cleaves N-glycosylated groups on proteins.

### Data Analysis and Statistics

All methods for data analysis of *in vivo* experiments are described in details in our recent publication (Joksimovic et al., 2020) and are only briefly described here. The data and statistical analysis comply with the recommendations on experimental design and analysis in pharmacology (Curtis et al., 2015, 2018). The declared group size is the number of independent values, and statistical analysis was done using these independent values. For each experiment, animals were randomly assigned to experimental groups in order to generate biological replicates, and the experimenter was blinded for the treatment until subsequent data analyses have been performed. No outliers were excluded in these experiments. For all studies animals were litter-matched and age-matched to keep the treatment groups as similar as possible. In order to assure stable recording conditions for the measurements of mechanical and thermal sensitivities, we determined baseline values on both paws on two days prior to injections of STZ and again prior to drug application. Each data point from experiments was expressed as mean ± SEM. Proper statistical analysis of the differences in effects between the treatment and the vehicle groups was performed using two-way RM ANOVA followed by Tukey's and Bonferroni's *post-hoc* tests and one-way RM ANOVA as appropriate. In multigroup studies with parametric variables, *post hoc* tests (recommended by GraphPad prism) were conducted only if F in ANOVA achieved the necessary level of statistical significance and there was no significant variance inhomogeneity. For *in vitro* experiments, data were analyzed with paired or unpaired *t*-test, as well with two-way RM ANOVA followed by appropriate *post-hoc* test. Significant differences between group means are indicated when *p* < 0.05. GraphPad Prism 7 (GraphPad Software, La Jolla, CA, USA) was used for all statistical analyses.

### Methodological Considerations

Our previous studies in rats used STZ-induced hyperglycemia to elicit type 1 PDN and investigated the role of Ca_V_3.2 channel up-regulation in boosting peripheral nociceptor excitability (Jagodic et al., 2007; Messinger et al., 2009; Choe et al., 2011; Obradovic et al., 2014) and glutamate release in the dorsal horn (Jacus et al., 2012). Here we use rats to demonstrate an effective anti-hyperalgesic action of i.t. injections of NEU with *in vivo* and to pursue molecular analysis of the channel protein. We also use mouse genetics to extend our findings and validate that Ca_V_3.2 channels are indeed the molecular substrates for the effects of NEU. In contrast, our biophysical studies, for practical reasons, were performed using recombinant human Ca_V_3.2 channels as in our previous study (Orestes et al., 2013). Although tissues from 3 different species were used, we believe our results are consistent because putative glycosylation sites are well-conserved in the human, rat and mouse Ca_V_3.2 channels.

## Results

### Effects of Enzymatic De-glycosylation on Ca_V_3.2 Current Amplitudes and Kinetics

**Figure 1:**
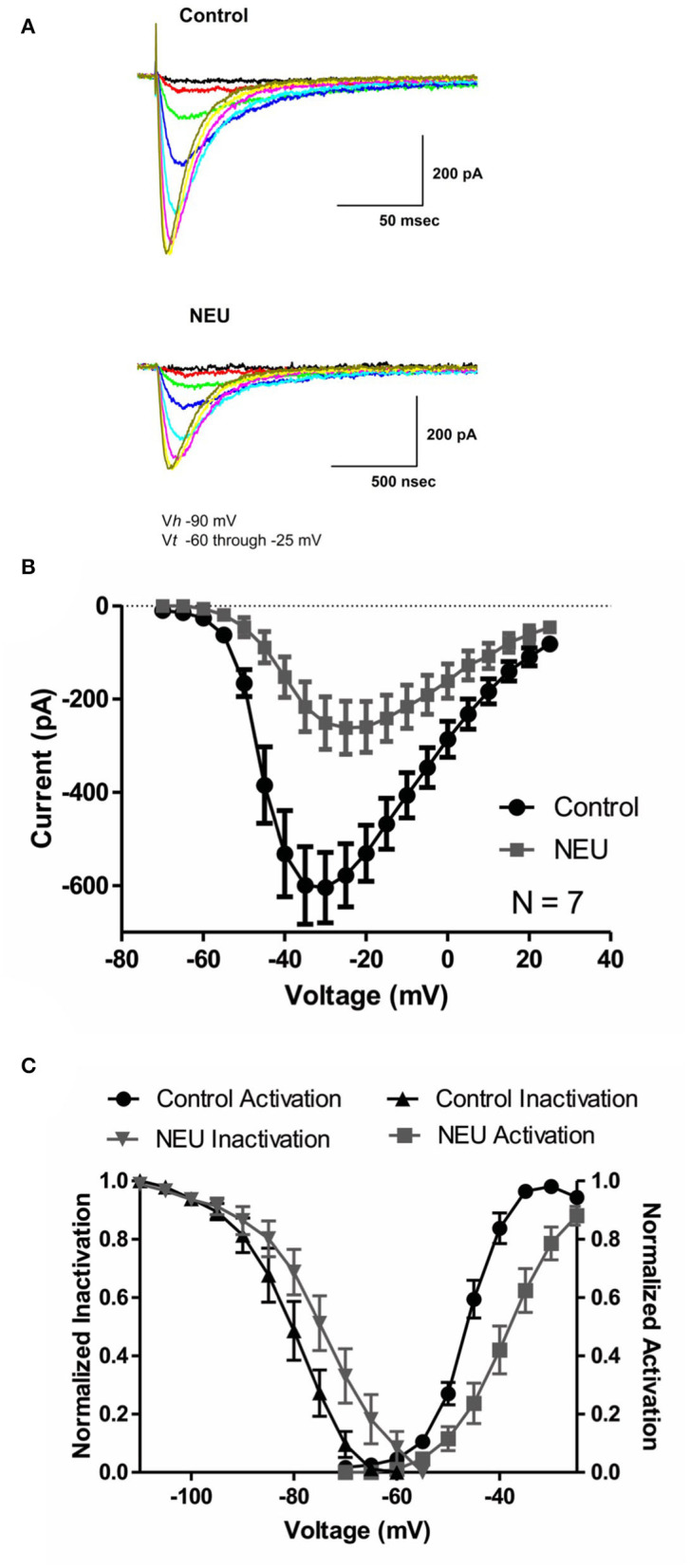
Effects of treatments with NEU (1.5 U/ml for 1–3 h) on current-voltage (I-V) relationships in HEK293 cells expressing stable Ca_V_3.2 channels grown in hyperglycemic cell culture medium. **(A)** Traces represent families of Ca_V_3.2 T-currents evoked in representative HEK293 cells in control (top panel) *vs*. treated (bottom panel) cohort by voltage steps from holding potential (V_h_) of −90 mV to test potentials (V_t_) from −60 to −25 mV in increments of 5 mV. Traces from two cells that were evoked at the same depolarizing test potentials are depicted in the same color. **(B)** Graph of I-V curves shows averages of peak currents at wide range of potentials from 7 cells in control untreated conditions (black symbols) and population of cell after treatment with NEU (gray symbols). Note that NEU treatment decreased peak currents over the range of test potentials more than 2-fold. **(C)** When compared to untreated control cells (black symbols) treatment of HEK293 cells with NEU (gray symbols) induced significant shift of V_50_ in steady-state inactivation curves from −79.7 ± 2.3 mV (*n* = 6) to −72.0 ± 2.4 mV (*n* = 9, *p* < 0.05). Similarly NEU induced depolarizing shift of V_50_ for steady-state activation from −45.7 ± 1.0 mV in control conditions to −37.9 ± 1.9 mV after NEU treatment (*n* = 7, *p* < 0.01). We used 2 mM Ca^2+^ as a charge carrier in these experiments.

We began our studies recording human recombinant Ca_V_3.2 currents expressed in HEK293 cells and determined the current-voltage (I-V) relationship in control (untreated) conditions and after treatment with NEU. Representative traces from these experiments (Figure 1A) and averaged current relationships (*n* = 7) from similar experiments (Figure 1B) are depicted and illustrate the effects of de-glycosylation. As expected from our previous work (Orestes et al., 2013), we confirmed that treatment with NEU (gray symbols) decreased peak T-current densities more than 2-fold over a broad range of test potentials when compared to T-currents recorded from control, untreated cells (black symbols). To determine if NEU treatment may also alter the voltage-dependence of T-channel gating (Figure 1C), we used peak current amplitudes elicited by voltage steps from holding potentials of −100 or −100 to −60 mV, to determine the voltage dependence of activation or steady-state inactivation, respectively (Figure 1C, right, left). When compared to untreated control cells (black symbols) treatment of HEK293 cells with NEU (gray symbols) induced a significant shift in the midpoint (V_50_) of steady-state inactivation from−80 mV (*n* = 6) to ~-71 mV (*n* = 9, *p* < 0.05). Similarly, NEU induced a depolarizing shift of V_50_ for steady-state activation from −46 mV in control conditions to −38 mV after treatment (*n* = 7, *p* < 0.01). Hence, NEU treatment significantly shifted to depolarizing potentials the voltage range predicted for steady-state current.

**Figure 2:**
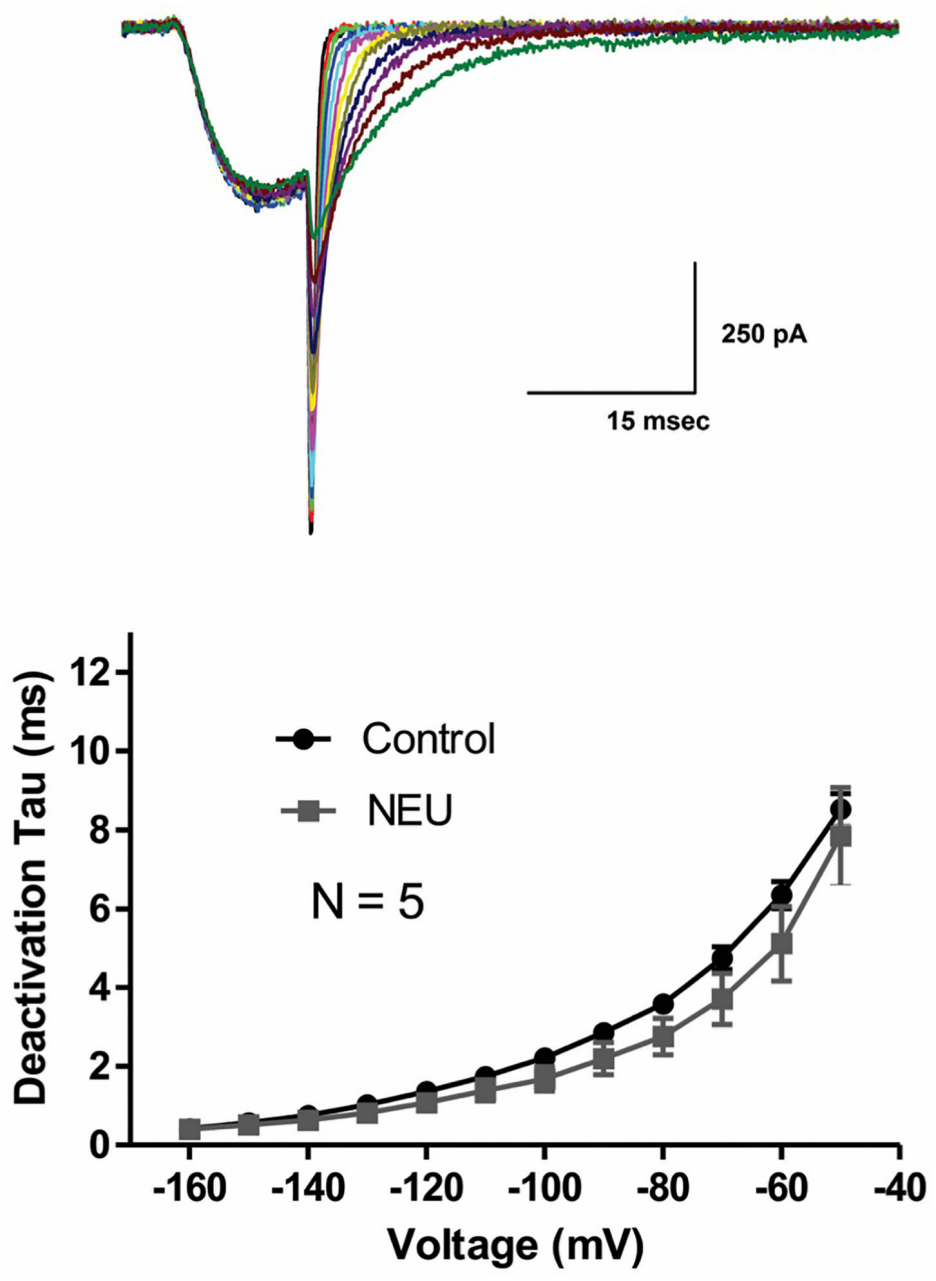
Minimal effect of NEU on deactivating Ca_V_3.2 current kinetics. Traces on the top of this figure are taken from a representative HEK293 cells expressing Ca_V_3.2 channels grown in hyperglycemic cell culture medium and treated with NEU for 1 h. Average graph from similar experiments in 5 untreated (control) cells and 5 treated cells is shown on lower panel of this figure. When compared with control conditions (black symbols) treatment with NEU (gray symbols) minimally affected time constant (tau) of deactivating tail current kinetics of recombinant Ca_V_3.2 currents. We used 2 mM Ca^2+^ as a charge carrier in these experiments.

Slow deactivation of T-channels allows calcium ions to enter the cell during the repolarizing phase of the action potential. To determine if NEU treatment may also further slow channel deactivation, we recorded deactivating T-currents in control conditions and after exposing HEK293 cells to NEU treatment. Representative current traces (Figure 2, top panel) and average data from similar experiments (Figure 2, bottom panel) indicate that deactivation time course of current decay was not significantly different between NEU-treatment (gray symbols) and control conditions (black symbols).

**Figure 3:**
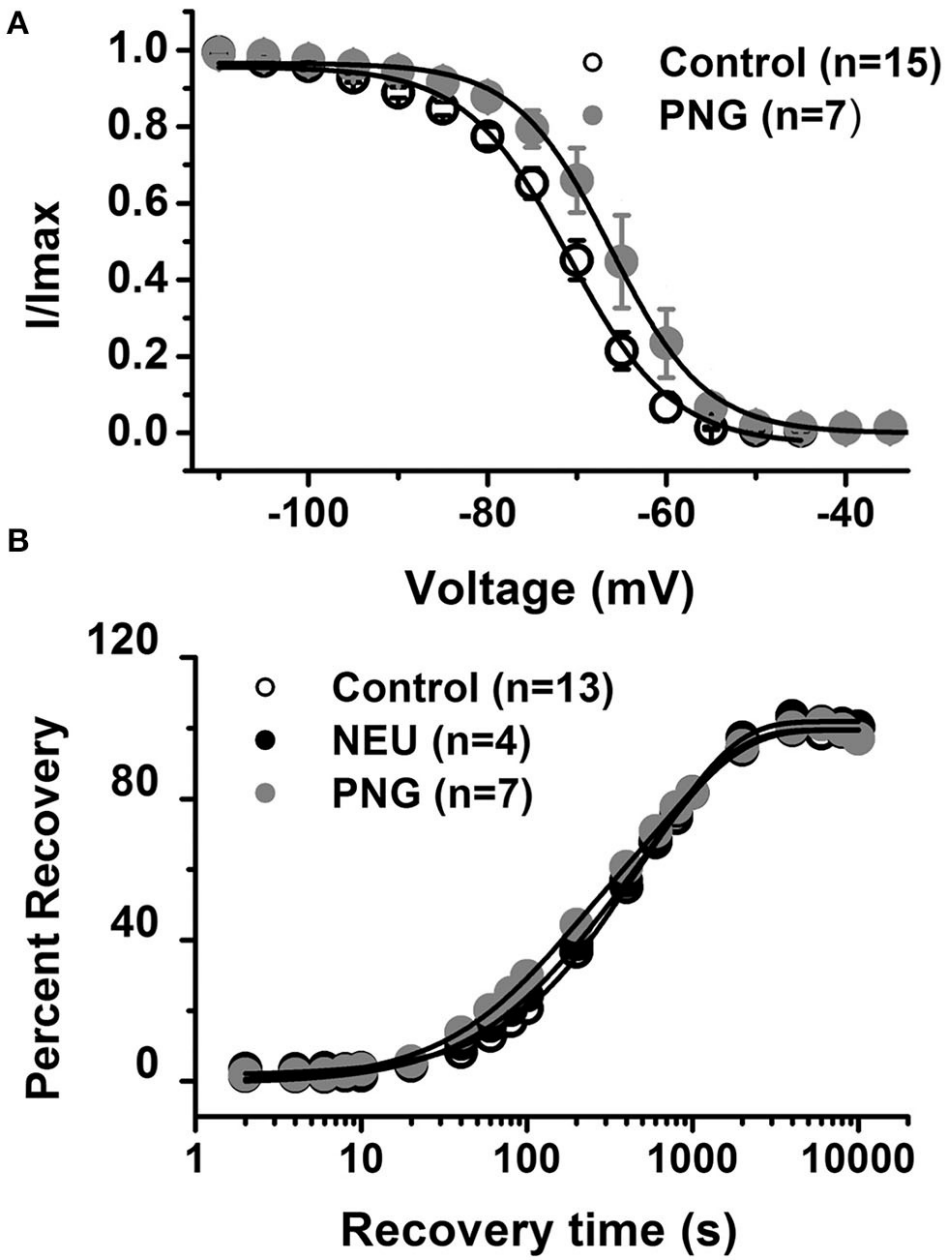
De-glycosylation with PNGase-F (PNG) causes depolarizing shift in voltage-dependent inactivation of Ca_V_3.2 currents similar to NEU, but neither PNG nor NEU influence time course of recovery from inactivation. **(A)** When compared to untreated control cells (open symbols) treatment of HEK293 cells with PNG (gray symbols) induced significant shift of V_50_ in steady-state inactivation curves from −71.6 ± 1.2 mV (*n* = 15) to −66.0 ± 2.2 mV (*n* = 7, *p* < 0.05). **(B)** Average graph from the experiments in 13 untreated (control) cells (open symbols), 4 cells treated with NEU (black symbols) and 7 cells treated with PNG (gray symbols) is shown on this panel. When compared with control conditions treatment with NEU or PNG minimally affected time course of recovery from inactivation of recombinant Ca_V_3.2 currents. We used 10 mM Ba^2+^ as a charge carrier in these experiments.

We previously reported that de-glycosylation of Ca_V_3.2 channels with PNG treatment decreased T-current densities similar to NEU (Orestes et al., 2013). To determine if PNG may also alter the voltage-dependence of inactivation of T-channels, we compared steady-state inactivation relationships determined with and without PNG-treatment. The average data points from the control conditions (open symbols) and PNG-treated cells (gray filled symbols), as well as best fits of these data are presented in Figure 3A. We found that, similar to NEU treatment, PNG induced a significant depolarizing shift in midpoint (V_50_) of steady-state inactivation, albeit smaller (about 6 mV; PNG *n* = 7, control *n* = 15, *p* < 0.05).

Finally, we compared the properties of recovery from inactivation in control untreated conditions (open symbols) with NEU-treatment (black filled symbols) and PNG-treated (gray filled symbols) cells (Figure 3B). These data show that there is no essential difference in the properties of recovery from inactivation after Ca_V_3.2 channels have been de-glycosylated. In summary, our biophysical studies indicate that de-glycosylation of Ca_V_3.2 channel had the biggest impact on T-current peak densities and voltage-dependent properties of channel gating whereas the kinetic properties of deactivation and recovery from inactivation were minimally affected.

### *In vivo* Treatment With NEU in the WT Mice Reversed Mechanical and Thermal Hyperalgesia Associated With STZ-Induced PDN

Our previous studies firmly established that Ca_V_3.2 isoform of T-channels is a major player in regulation of sensory neuron excitability (Nelson and Todorovic, 2006; Orestes et al., 2013; Joksimovic et al., 2018). Furthermore, it is generally accepted that changes in excitability of sensory neurons can directly influence pain thresholds (Campbell and Meyer, 2006). Hence, we reasoned that de-glycosylation of Ca_V_3.2 channels may decrease sensory neuron excitability and consequently reverse diabetes-induced hyperalgesia due to decreased T-current densities and a depolarizing shift in the voltage range predicted for steady state window current. We tested this idea by injecting NEU into the plantar surface of the paw (i.pl.) in the peripheral receptive fields of WT mice and measuring thermal and mechanical nociceptive responses after rendering animals hyperglycemic following i.p. injections of STZ.

**Figure 4:**
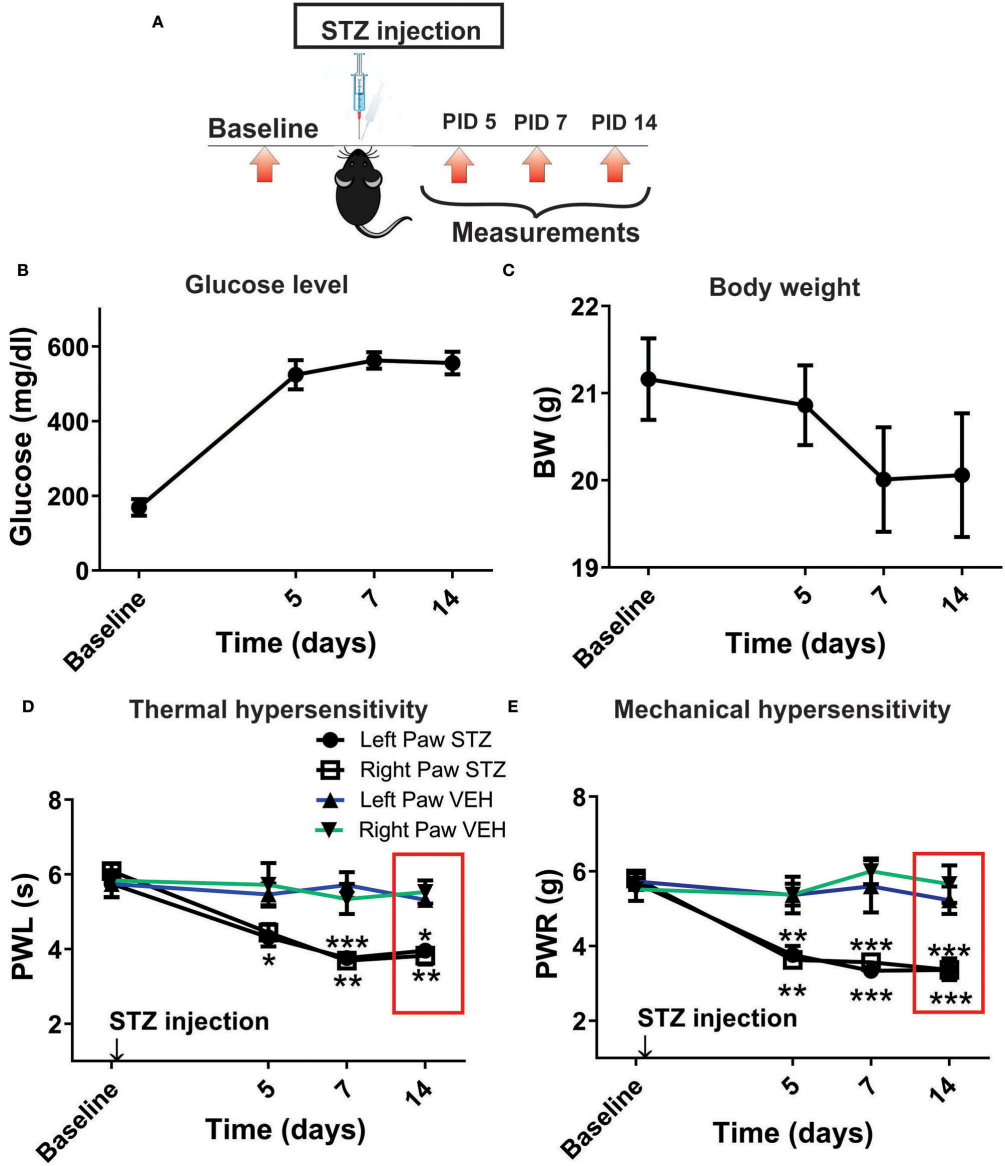
Streptozotocin-induced diabetes is accompanied with thermal and mechanical hypersensitivity in WT mice. **(A)** Experimental protocol for injection of STZ and time points of measurement of threshold to thermal and mechanical stimuli. **(B)** Time course of the development of hyperglycemia in mice after STZ injection (*n* = 10). **(C)** Changes in body weight of mice injected with STZ (*n* = 10, One way RM ANOVA, *p* = 0.244). **(D)** Time course of the development of hypersensitivity to thermal stimulus in mice after STZ injection (*n* = 10, Two-way RM ANOVA, treatment factor significant *p* < 0.001; *F*_(3.28)_ = 24.54; Tukey *post-hoc* test: *p < 0.05; **p < 0.01; ***p < 0.001). **(E)** Time course of the development of hypersensitivity to mechanical stimulus in mice after STZ injection (*n* = 10, Two-way RM ANOVA, treatment factor significant *p* < 0.001; *F*_(3,28)_ = 30.14; Tukey *post-hoc* test: **p < 0.01; ***p < 0.001).

**Figure 5:**
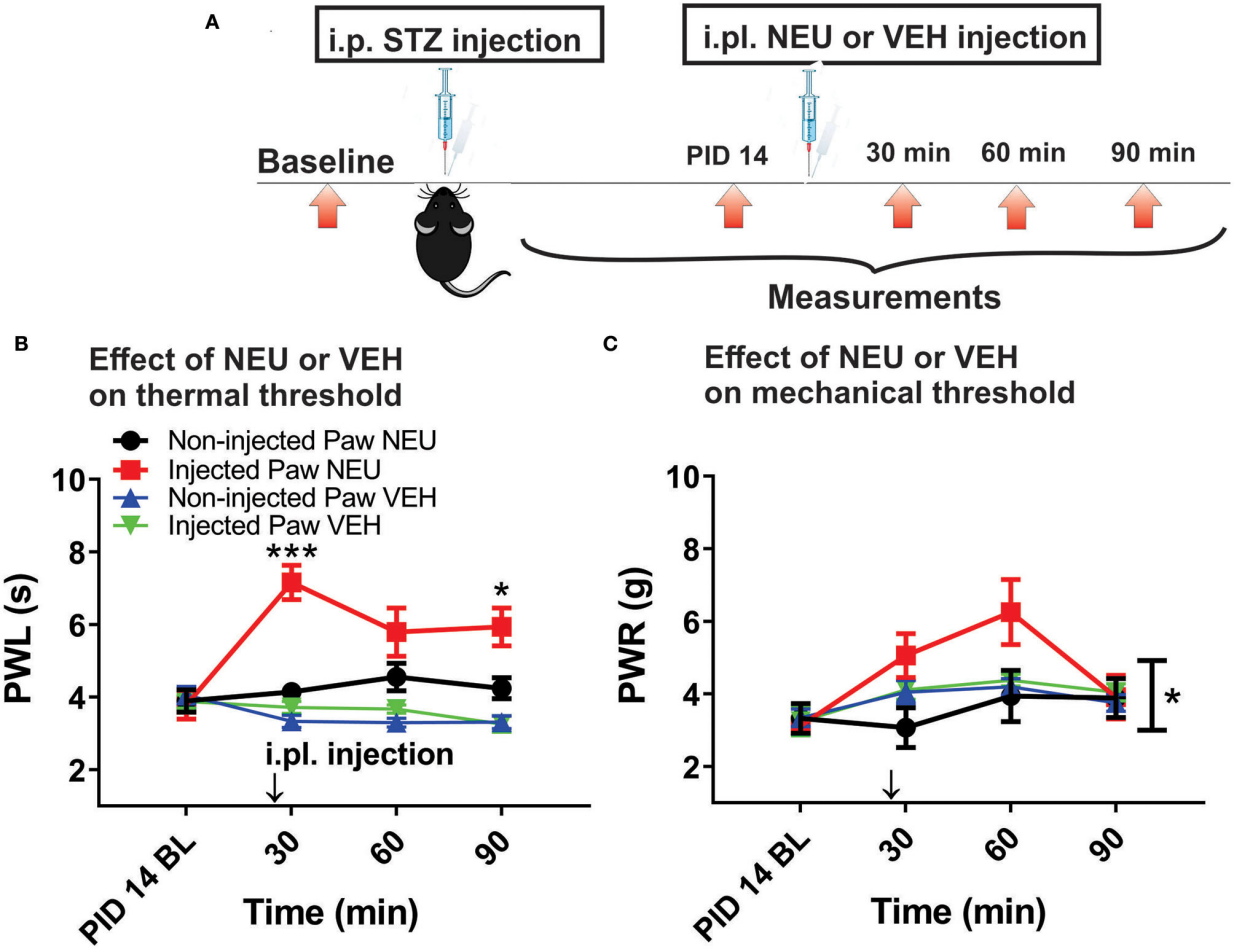
Effects of intraplantar (i.pl.) NEU on thermal and mechanical hypersensitivity in diabetic WT mice. **(A)** Experimental protocol of i.pl. injection of NEU and time points of measurement of threshold to thermal and mechanical stimuli. **(B)** Time course of thermal antihyperalgesic effect of intraplantar NEU or VEH injected unilaterally in diabetic mouse paw (*n* = 6 NEU group, Two-way RM ANOVA, treatment factor significant *p* < 0.001, *F*_(1,14)_ = 35.32; Sidak *post-hoc* test: *p < 0.05; ***p < 0.001; *n* = 4 VEH group). **(C)** Time course of mechanical antihyperalgesic effect of intraplantar NEU or VEH injected unilaterally in diabetic mouse paw (*n* = 5 NEU group, *p < 0.05 Two-way RM ANOVA, treatment factor significant *p* =0.036, *F*_(1,8)_ = 6.33; *n* = 6 VEH group).

Our previous study established that local i.pl. injections of NEU effectively reversed thermal and mechanical hyperalgesia in diabetic ob/ob mice as a representative animal model of Type 1 PDN (Orestes et al., 2013). Here, we investigated if the same approach is effective in mice with STZ-induced Type 2 painful PDN. We used for these *in vivo* studies 2 cohorts of diabetic mice injected with STZ i.p. and followed them for two weeks: C57Bl/6j wild type (WT) mice and Ca_V_3.2 knock-out (KO mice). Figure 4A shows the experimental protocol for the induction of diabetes with single STZ injections in mice. The onset of hyperglycemia was monitored by measuring glucose levels on post-STZ injection (PID) days 5, 7 and 14 (Figure 4B). All animals injected with STZ developed equally severe hyperglycemia as determined by daily measurements of blood glucose that ranged from 400 to 600 mg/dl. Since animals ordinarily may exhibit fluid loss and decreased food intake from hyperglycemia, we also regularly followed animal body weights (BW) and found that they did not significantly differ from the baseline values (One way ANOVA, p = 0.2981) obtained before STZ injections (Figure 4C). Hyperalgesia development was determined by measuring thresholds to thermal (Figure 4D) and mechanical (Figure 4E) stimuli on PID 5, 7 and 14. Note that stable thermal and mechanical hyperalgesia developed after 7 days post STZ injections as evidenced by about 30–40% decrease in PWL and PWR values, respectively. All local i.pl. injections of NEU or vehicle were performed 2-weeks following STZ injections (Figure 5A). Either 10 μl of 1.5 U/cc NEU or vehicle (saline) (Figures 5B,C) was injected i.pl. in the right hind paws of adult female diabetic mice 14 days post-STZ. This concentration of NEU was the same as one that we used for our biophysical *in vitro* studies. We then measured thermal PWLs and mechanical PWRs in both left (control paws, black symbols) and right (injected paws, red symbols) at 30, 60, and 90 min after injection. Note that diabetic WT mice have decreased baseline PWLs and PWRs post-STZ injections indicating prominent thermal and mechanical hyperalgesia (Figures 5B,C, respectively). We found that single i.pl. injections of NEU, but not VEH, produced a lasting decrease in sensitivity to thermal stimuli in diabetic WT mice at all measured time points (Figure 5B; right paws vs. left paws: ^***^p < 001; ^*^p < 0.05; *n* = 6; two-way ANOVA with repeated measures). This was manifested by prolongation, by about 30–60%, in PWLs in the injected paws indicating complete return to pre-STZ baseline values. Of note, PWLs in uninjected (left) paws remained decreased throughout testing, indicating a lack of systemic effect. We previously demonstrated using identical protocol that injection of this concentration of NEU i.pl. did not have a significant antinociceptive effect on thermal PWLs, however it exerted mild but significant increase in paw withdrawal responses to Von Frey filament in healthy WT mice (Orestes et al., 2013). Transient hyperalgesia noted after injection could be due to intraplantar injection procedure itself, or even due to potential effects of NEU on other targets, the effects of NEU that yet to be investigated. Similarly, we found that single i.pl. injections of NEU, but not VEH, produced a lasting overall decrease in sensitivity to mechanical stimuli in diabetic WT mice with nearly complete reversal of diabetic hyperalgesia at the time point of 60 min post-STZ injections (Figure 5C; *n* = 5, right paws vs. left paws ^*^p < 0.05). An overall statistical significance of the treatment factor was represented over time, and not in a specific time point, because the interaction between two factors in two way ANOVA analysis was not significant and therefore we did not use the *post-hoc* analysis to further determine the significance per time point.

**Figure 6:**
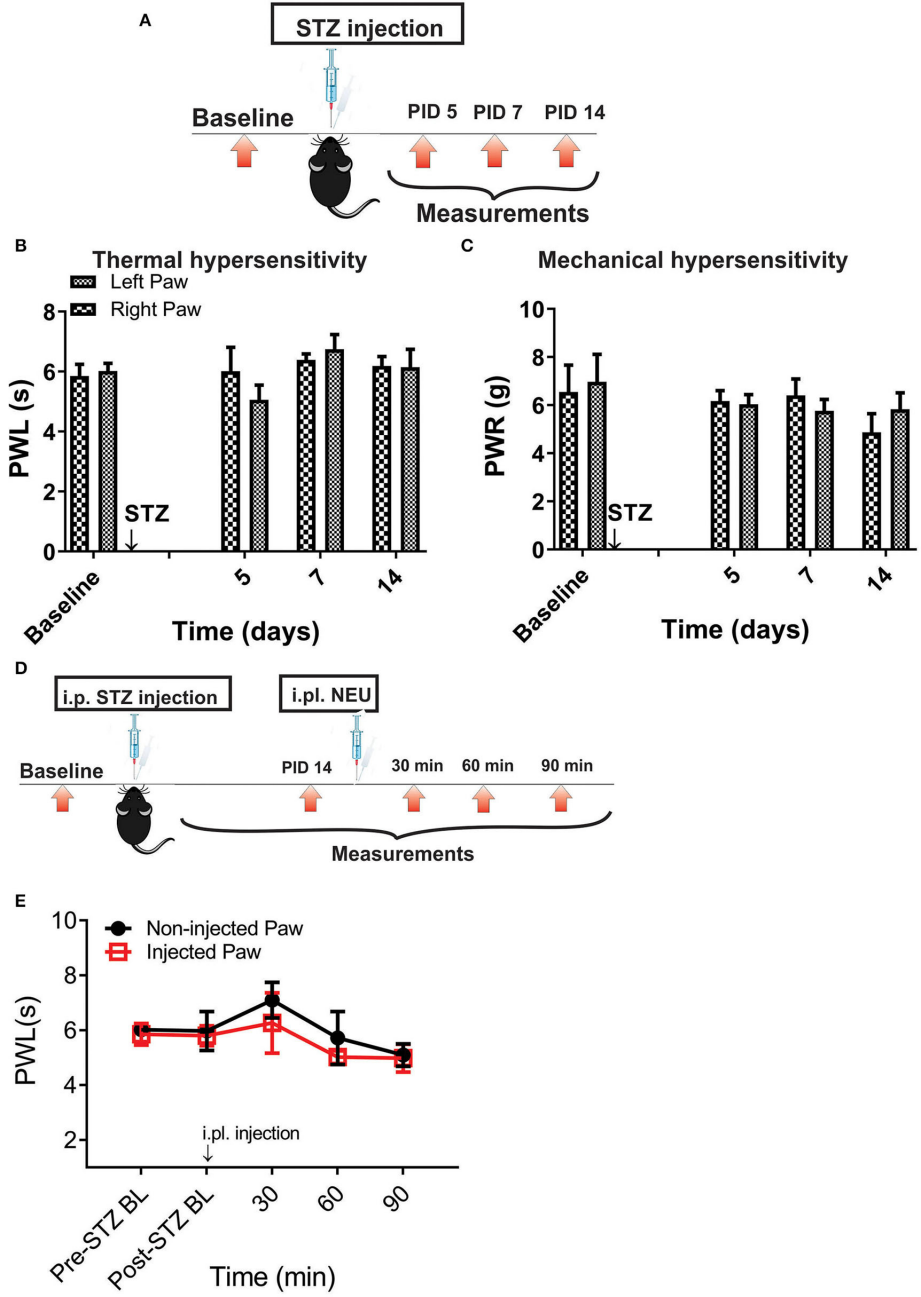
Thermal and mechanical hypersensitivity failed to develop in streptozotocin-induced diabetes in Ca_V_3.2 KO mice. **(A)** Experimental protocol for injection of STZ and time points of measurement of threshold to thermal and mechanical stimuli. **(B)** Lack of development of hypersensitivity to thermal stimulus in mice after STZ injection (*n* = 4). **(C)** Lack of development of hypersensitivity to mechanical stimulus in mice after STZ injection (*n* = 4). **(D,E)** To test whether the selective anti-hyperalgesic effect of NEU in diabetic WT mice is related to its effects on Ca_V_3.2 channels, we injected 1.5 U/cc of NEU i.pl. in the right paws (arrow) of Ca_V_3.2 KO mice (*n* = 4) and found that it did not affect thermal PWLs (Two-way RM ANOVA, treatment factor *p* > 0.05, right vs. left paws) up to 90 min after injection.

**Figure 7:**
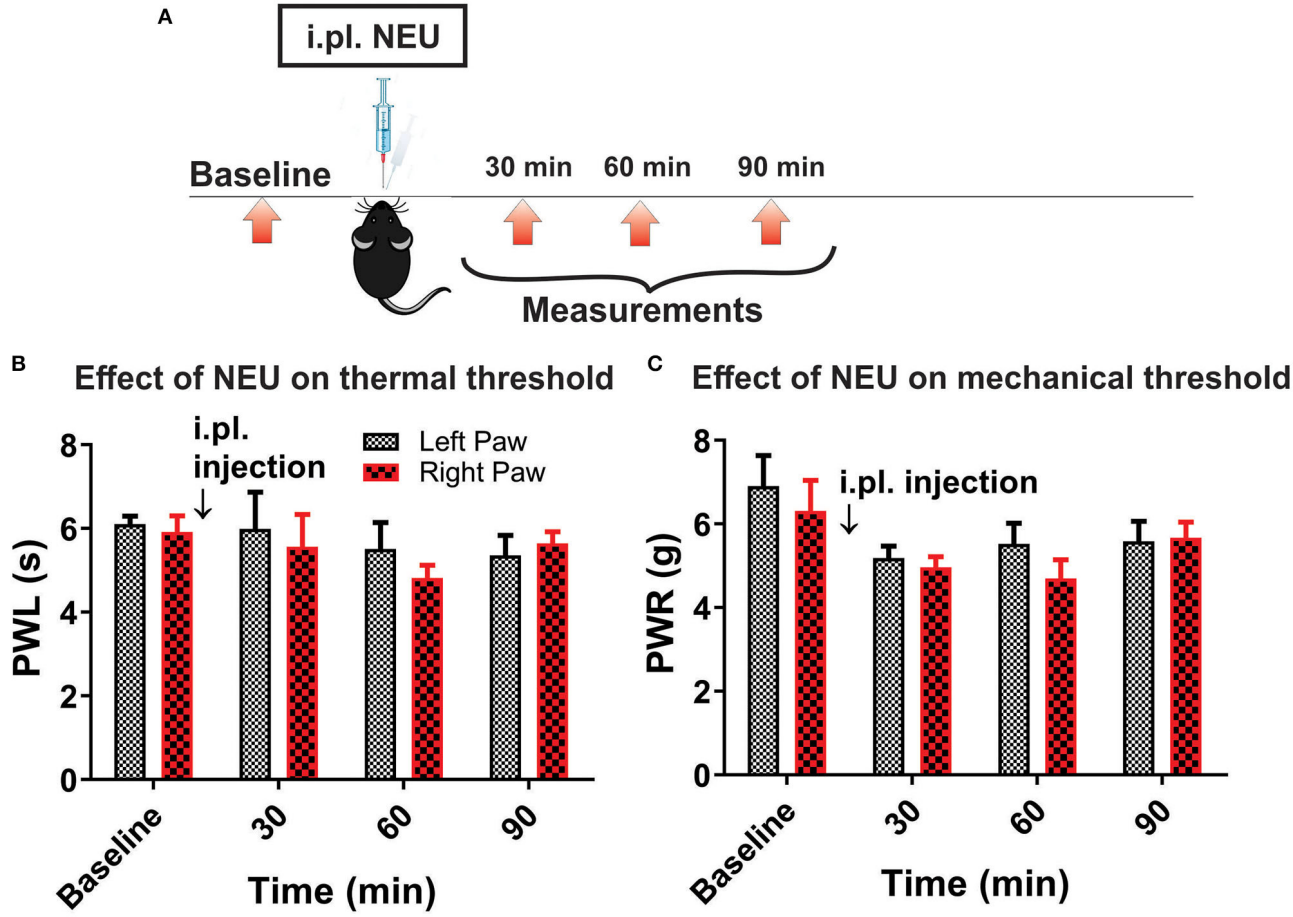
Lack of effect of intraplantar NEU on thermal and mechanical thresholds in Ca_V_3.2 KO mice. **(A)** Experimental protocol for intraplantar injection of NEU and time points of measurement of threshold to thermal and mechanical stimuli. **(B)** Lack of thermal analgesic effect of intraplantar NEU injected unilaterally in healthy Ca_V_3.2 KO mouse paw (*n* = 6, Two-way RM ANOVA, treatment factor *p* = 0.51). **(C)** Lack of mechanical analgesic effect of intraplantar NEU injected unilaterally in healthy Ca_V_3.2 KO mouse paw (*n* = 6, Two-way RM ANOVA, treatment factor *p* = 0.42).

However, it is possible that effect of NEU *in vivo* may not be related only to its effects on Ca_V_3.2 channels in sensory neurons. Hence, we used mouse genetics in order to evaluate the importance of the Ca_V_3.2 channel as a molecular target for effective NEU-induced reversal of hyperalgesia in PDN. We have followed the same protocol of STZ administration in Ca_V_3.2 KO mice and thermal and mechanical threshold measurements as in WT mice (Figure 6A). Similarly to our previous finding (Latham et al., 2009), we confirmed that diabetic Ca_V_3.2 KO mice (with fasting glucose levels already on Day 7 at 600 mg/dl) failed to develop thermal and mechanical hyperalgesia after STZ injections and hence, demonstrated similar values of baseline PWLs and PWRs before and after injections of STZ (Figures 6B,C, *p* > 0.05). Importantly, i.pl. injections of NEU did not exhibit any effect on thermal nociception in diabetic Ca_V_3.2 KO mice injected with STZ (Figures 6D,E). To test further if anti-hyperalgesic effect of NEU in WT mice is related to its effects on Ca_V_3.2 channels, we injected 1.5 U/cc of NEU i.pl. in the right paws (arrow) of Ca_V_3.2 KO mice (Figure 7A). We found that NEU did not affect baseline thermal or mechanical thresholds (*p* > 0.05, right vs. left paws) up to 90 min after injection in mutant mice (Figures 7B,C, respectively). This strongly suggests that NEU is devoid of any off target effects and that the anti-hyperalgesic effect exhibited after NEU application can be attributed to effects of NEU against Ca_V_3.2 channels in peripheral nociceptive nerve endings.

### Intrathecal Administration of Neuraminidase Completely Reversed Thermal Heat Hyperalgesia in STZ-Treated Diabetic Rats

**Figure 8:**
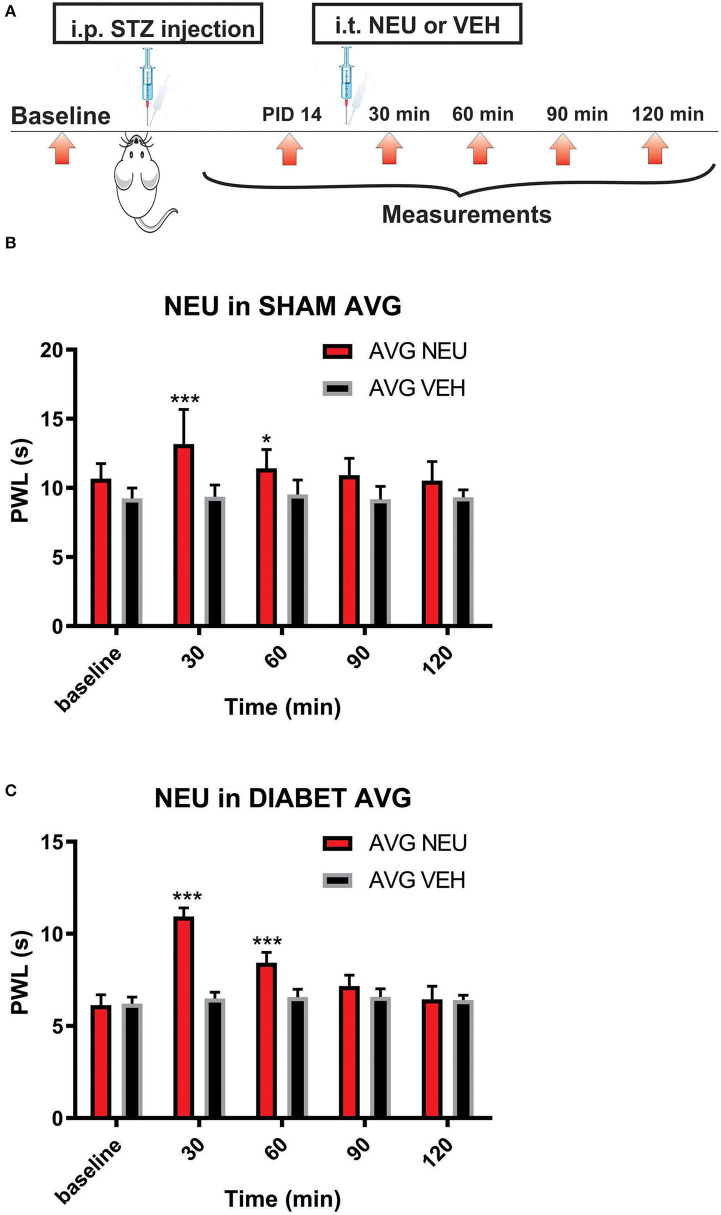
Intrathecal (i.t.) NEU reduces thermal hyperalgesia in both healthy and diabetic rats. **(A)** Experimental protocol for i.t. injection of NEU and time points of measurement of threshold to thermal stimulus. **(B)** Time course of thermal antihyperalgesic effect of intrathecal NEU in healthy rats (*n* = 9, Two-way RM ANOVA, treatment factor significant *p* = 0.0022, *F*_(1,14)_ = 14; Bonferroni *post hoc* test: *p < 0.05; ***p < 0.001). **(C)** Time course of mechanical antihyperalgesic effect of intrathecal NEU in diabetic rats (*n* = 9, Two-way RM ANOVA, treatment factor significant *p* < 0.001; *F*_(1,15)_ = 74.8; Bonferroni *post hoc* test:; ***p < 0.001). The y axis represents average thresholds to the stimulus (AVG).

**Figure 9:**
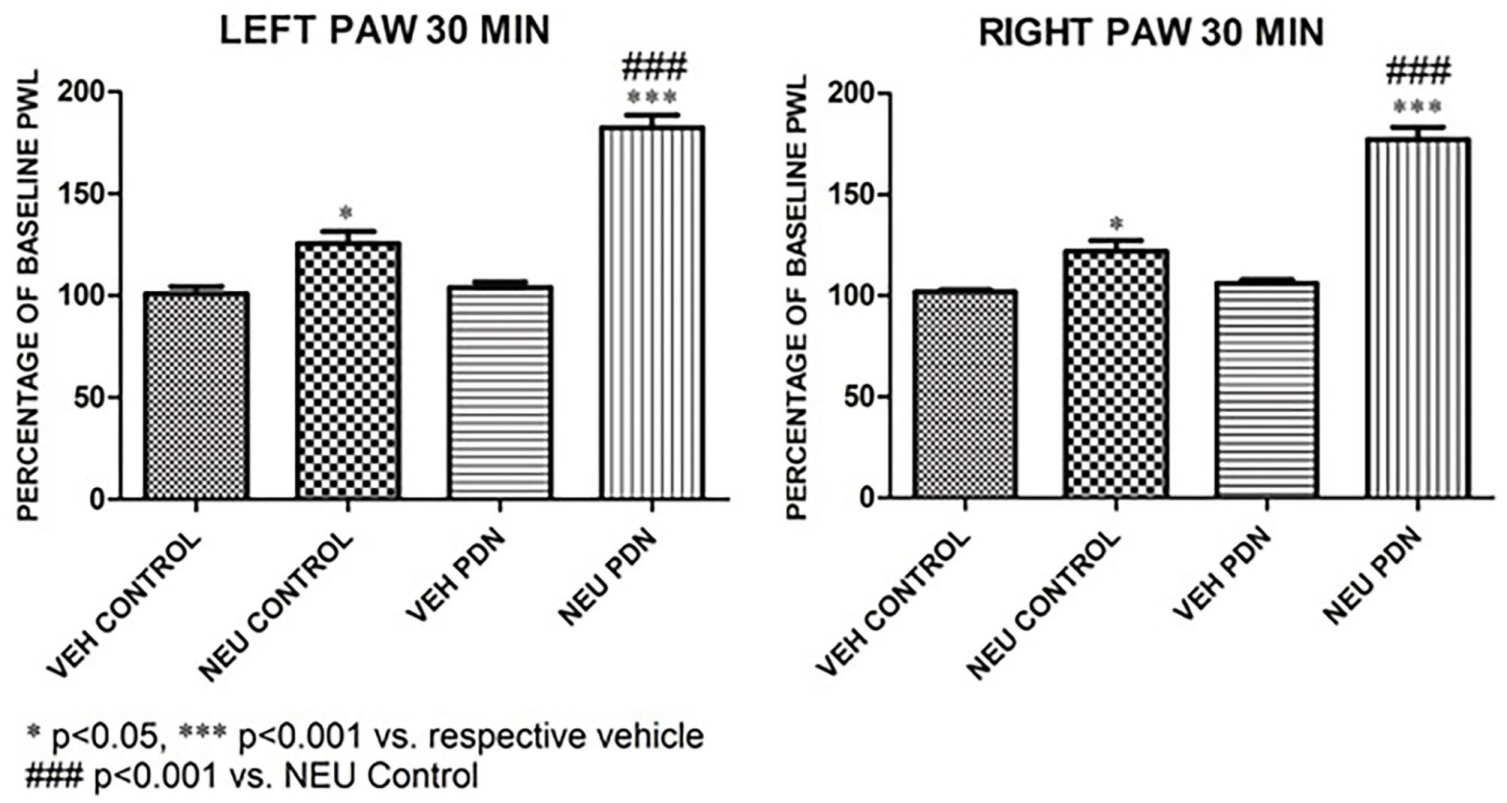
Intrathecal (i.t.) injections of NEU induced more prominent effect on thermal nociception in the diabetic than in healthy rats. The presented data are from the same experiments depicted on Figure 8 except that they are normalized to the baseline PWLs before i.p. injections of STZ or vehicle. Average graphs demonstrate that NEU effectively increased thermal PWLs in both paws of diabetic (PDN) rats while having a much smaller effect on healthy (control) rats as determined 30 min following i.t. injection (*p* < 0.001). Notably, when compared to i.t injections of vehicle (VEH), i.t. injections of NEU induced about 3-fold higher increase in PWLs in diabetic (82 ± 6% for left paws, and 77 ± 6% for right paws, *p* < 0.001, *n* = 7) than in control group (25 ± 6% for left paws, and 22 ± 6 % for right paws, *p* < 0.001, *n* = 9).

Our presented data with i.pl. injections support the idea that de-glycosylation of Ca_V_3.2 channels in peripheral nociceptive endings reverse STZ-induced hyperalgesia. However, we previously demonstrated that presynaptic Ca_V_3.2 channel in both rat DRG neurons (Jagodic et al., 2007; Messinger et al., 2009) and rat dorsal horn of the spinal cord (Jacus et al., 2012) are upregulated in STZ-induced PDN. Hence, we tested the hypothesis that intrathecal (i.t.) injections of NEU that target nociceptive neurons in both spinal dorsal horn and DRG (Messinger et al., 2009) could reverse hyperalgesia to noxious heat in diabetic rats (Figure 8A). Rats that were injected with vehicle (saline) i.p. were used as sham controls (Figure 8B). Rats having an average blood glucose reading 410 ± 73 mg/dl were considered hyperglycemic and were included in the experimental group 14 days following injections of STZ (Figure 8C). As presented on Figure 8, we noticed that only rats injected with STZ developed stable heat hyperalgesia (about 30–40% decrease in paw withdrawal latency (PWLs) in both paws (Figure 8C). Importantly, when NEU (1.5 U/cc) was injected i.t. in a total volume of 50 μL normal saline after brief isoflurane anesthesia, it effectively increased thermal PWLs in paws of diabetic rats (Figure 8C) while having significant but smaller effect on sham (control) rats (Figure 8B) as determined 30 and 60 min following i.t. injection. Notably, when compared to the normalized baseline PWLs, i.t. injections of NEU induced a roughly 3-fold higher increase in PWLs in the diabetic (NEU PDN, 77 ± 6 %, *n* = 7) than in the sham group (NEU CONTROL, 25 ± 6%, *n* = 9) at the time point of 30 min post-injection (*p* < 0.001, two-tailed unpaired *t*-test, Figure 9).

### Gel-Shift Analysis of Ca_V_3.2 Proteins in the DRG Tissues From Healthy and STZ-Treated Diabetic Animals

Our *in vivo* data presented on Figures 8, 9 show that STZ-treated rats exhibited an increase in averaged thermal PWLs after i.t. injection of NEU as compared to the group that received vehicle. This strongly suggest that glycosylated Ca_V_3.2 channels in the dorsal horn and DRGs may contribute to the development of hyperalgesia in diabetic animals. Hence, we investigated the possibility that in rat nociceptors Ca_V_3.2 channels are glycosylated. Ca_V_3 isoforms contain multiple potential N-X-S/T motifs for potential N-linked glycosylation in their extracellular loops. To test the idea that recombinant Ca_V_3.2 channels may be glycosylated at critical asparagine (Asn, N) residues in these N-X-S/T motifs, we obtained in our previous study single-point mutants of Ca_V_3.2 in which these Asn resides were mutated into glutamines (Q), namely N192Q, N1466Q, and N271Q (Orestes et al., 2013). Indeed, in that study we identified N192 and N1466 as important regulators of Ca_V_3.2 channel activity and membrane expression, respectively (Orestes et al., 2013). However, the state of glycosylation of Ca_V_3.2 channels in their native environment in DRG in physiological and pathological conditions associated with PDN is not known. Here, we took advantage of the gel-shift analysis to determine the degree of glycosylation of Ca_V_3.2 channels in DRG tissues since de-glycosylation of proteins can result in a faster electrophoretic mobility, and thus a lower apparent molecular weight (MW).

**Figure 10:**
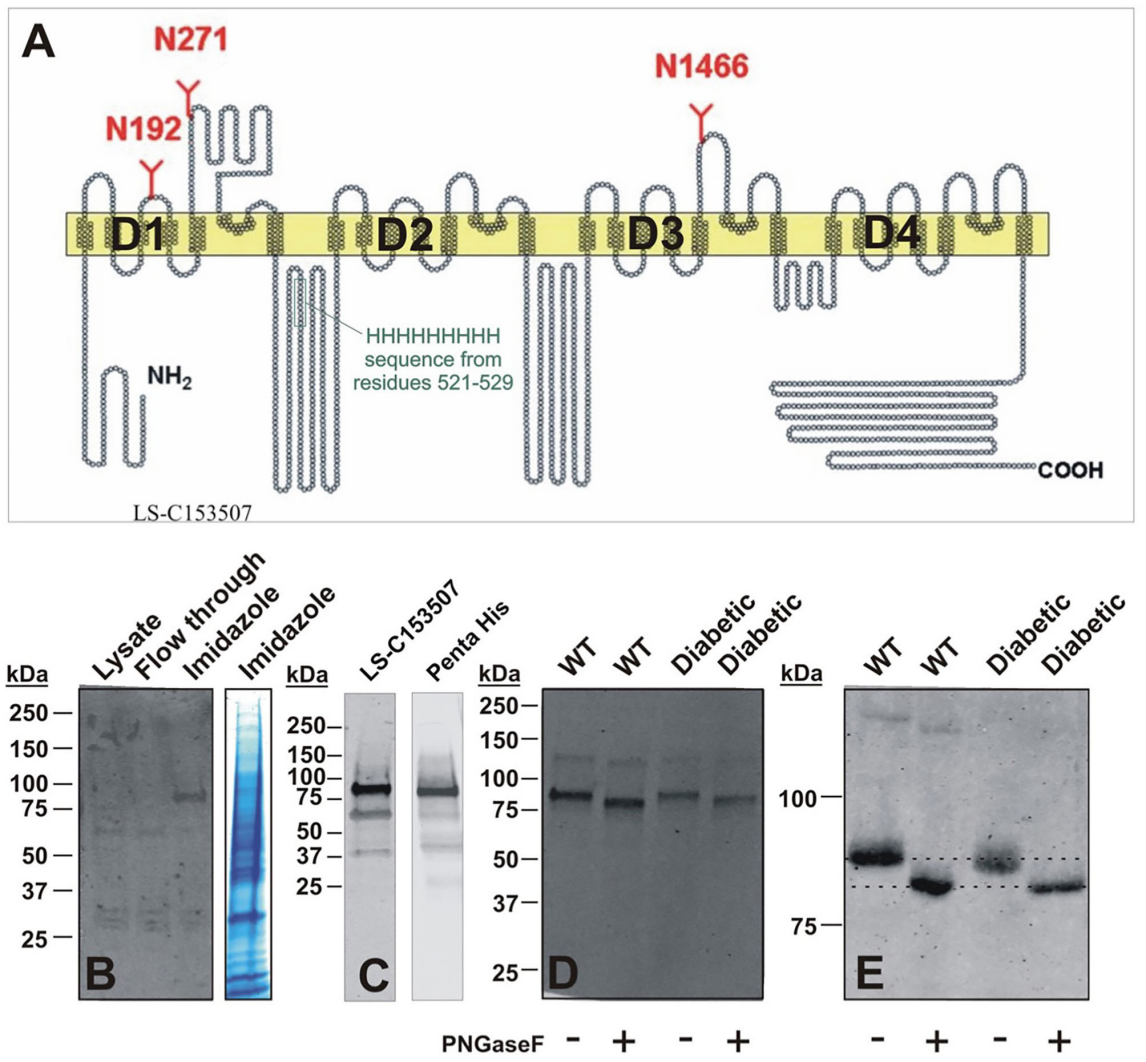
Molecular basis of glycosylation of native Ca_V_3.2 channels in rat DRG. **(A)** Hydropathy plot of the Ca_V_3.2 channel, illustrating glycosylation sites and native nona-histidine motif. **(B)** Purification of nona-histidine containing fragments of Ca_V_3.2 by Ni-NTA chromatography; left panel is immunoblot with LS-C153507 and right panel is stained with Simply Blue. Note that in the left panel there is a specific immunoreactive band in the imidazole elution lane between 75 and 100 kDa that is not found in the lysate and flow through. **(C)** Specificity of LS-C153507 demonstrated by anti-Penta His antibody that showed virtually identical immunoreactive band with MW between 75 and 100 kDa. **(D)** Separation of PNGaseF treated imidazole elutions in **(B)** by 4–20% SDS-PAGE strongly suggests N-terminal Ca_V_3.2 fragments are glycosylated in rat DRGs. **(E)** Separation of PNGaseF treated imidazole elutions in **(B)** by 6% SDS-PAGE confirms glycosylation of N-terminal Ca_V_3.2 fragments as detected by gel-shift in both WT healthy and diabetic rats.

Interestingly, two putative glycosylation sites (N192 and N271) are located in the repeat D1 of Ca_V_3.2 channels (Orestes et al., 2013). To purify native DRG Ca_V_3.2 channels using immobilized metal affinity chromatography (IMAC), we took advantage of the unique nine histidine repeat (non-ahistidine motif amino acids 521-529) in D1-D2 cytoplasmic loop of Ca_V_3.2 channels (Figure 10A). Lumbar DRGs were harvested from healthy, sham injected or diabetic STZ-injected rats (two per each experiment) as described in our recent publication (Joksimovic et al., 2018). N-terminal fragments of the Ca_V_3.2 channel containing the nona-histidine motif were captured using Ni-NTA (Qiagen) beads added directly to the RIPA cell lysate following syringe homogenization. After a 2 h incubation, the Ni-NTA beads were washed. Proteins that remained bound to the Ni-NTA beads were eluted with 200 mM imidazole in Ni-NTA wash buffer. De-glycosylation was performed with 500 units of PNGase-F (45 min on room temperature). After transfer, membranes were blocked (TBST 5% milk), and the polyclonal antibody LS-C153507 (LifeSpan BioSciences) was used to detect N-terminal domain of Ca_V_3.2 encompassing residues 185-253. Alternatively, the anti-Penta His antibody (Qiagen) was used to probe for the nona-histidine motif in Ca_V_3.2. Blots were incubated with fluorescent IRDye 800CW donkey anti-rabbit (LS-C153507), or IRDye 800CW Donkey anti-mouse (Penta His) secondary antibodies for fluorescence visualization detected with an Odyssey imager (Li-Cor).

Purification of nona-histidine containing fragments of Ca_V_3.2 by Ni-NTA chromatography is depicted on Figure 10B, with the left panel showing an immunoblot with N-terminal anti-Ca_V_3.2 antibody (LS-C153507) and right panel is stained with Simply Blue. Note that in the left panel there is a specific immunoreactive band in the imidazole elution lane of about 90 kDa that is not found in the lysate or flow through. Figure 10C demonstrates an independent identification of the N-terminal fragment of Ca_V_3.2 using the anti-Penta His antibody. LS-C153507 immunoreactivity as described in panel 10C is shown in the left panel; the right panel illustrates immunoreactivity of a protein band of the same molecular weight of about 90 kDa using the anti-Penta His antibody. Separation of PNG-treated imidazole elutions in Figure 10D by 20% SDS-PAGE and in Figure 10E by 6% SDS-PAGE confirms glycosylation of N-terminal Ca_V_3.2 fragments in tissue homogenates from healthy sham and diabetic STZ-treated cohorts as evidenced by shift of the bands in the presence of PNG of about 5 kDa. We found a small but consistent shift in apparent MW of N-terminal fragment of Ca_V_3.2 in all 5 experiments with samples from sham and diabetic animals. Overall, PNG treatment induces an average shift in bands in the sham group of 4.5 ± 0.5 kDa, as well as in the diabetic group of 4.5 ± 0.7 kDa (*p* = 0.98). We conclude that N-terminal fragments of native Ca_V_3.2 channels in DRGs are glycosylated in both diabetic and in the healthy animals.

## Discussion

### Glycosylation of Ca_V_3.2 Channels Contributes to Painful PDN

Our results presented herein using STZ injections and mouse genetics complement our earlier study with ob/ob mice (Orestes et al., 2013) and demonstrate that glycosylation-induced alterations in Ca_V_3.2 channels in sensory neurons *in vivo* may directly influence diabetic hyperalgesia. Furthermore, we demonstrated in this study that enzymatic glycosylation inhibitors such as NEU can be used to ameliorate painful symptoms of PDN in Type 1 diabetes. However, we need to keep in mind that multiple pathogenic mechanisms may contribute to the impaired function of ion channels in sensory neurons of animals with PDN. For example, formation of metabolites of glucose such as intracellular advanced glycation end products (AGE), inflammatory cytokines, increased aldose reductase activity, oxidative stress, and methylglyoxal modification have been implicated in various studies (Edwards et al., 2008; Bierhaus et al., 2012; Eberhardt et al., 2012; Jack and Wright, 2012). However, in several preclinical and clinical studies, targeting many of these mechanisms has not provided complete pain relief in PDN (Obrosova, 2009). Inconsistent responses to current therapies strongly suggest that a number of different mechanisms may contribute to symptoms of painful PDN. We have previously demonstrated that direct selective Ca_V_3.2 channel blockers and *in vivo* silencing of Ca_V_3.2 channel in DRG neurons effectively reverse pain in animal models of Type 1 and Type 2 diabetes (Latham et al., 2009; Messinger et al., 2009; Choe et al., 2011; Obradovic et al., 2014). Prevailing literature has focused on glycation, the intracellular accumulation of AGE on arginine and lysine residues, as the non-enzymatic modification of proteins in a diabetic environment (Jack and Wright, 2012). To our knowledge the effects of enzymatic process such as glycosylation on extracellular asparagine residues of Ca_V_3.2 channels on painful PDN have not been reported in other studies. Our studies are largely in agreement with *in vitro* molecular studies by Weiss and Zamponi (2013), Lazniewska and Weiss (2016) and together provide important conceptual advances in the field of pain research and PDN by focusing on an under-appreciated mechanism, specifically post-translational Ca_V_3.2 channel modification of critical asparagine residues by glycosylation. Here, we propose that signaling pathways involving post-translational modification of Ca_V_3.2 channels *via* glycosylation contribute to alterations of Ca_V_3.2 current kinetics and likely influence the ensuing hyperalgesia and allodynia in PDN. It is well-known that N-glycosylation, similarly to other post-translational modifications such as de-ubiquitination (García-Caballero et al., 2014; Weiss and Zamponi, 2017; Joksimovic et al., 2018), of calcium channels can affect trafficking of the channel. In fact, N-glycosylation is essential for T-channel membrane expression. Therefore, we posit that a therapeutic strategy to suppress pain by normalizing nociceptive channel function (i.e., reversing or preventing excessive glycosylation of Ca_V_3.2 channel) will be advantageous to that of direct channel blockade because by correcting the pathology of PDN at its source, this approach should avoid the multitude of unintended side effects that are associated with current therapies. It is hoped that our studies may establish the role of glycosylation in regulating the function of Ca_V_3.2 and other nociceptive ion channels in sensory neurons both under normal conditions and in pathologies associated with PDN. We envision that specific targeting of glycosylation pathways of Ca_V_3.2 channels may be developed into a novel, effective and safe mechanistic-based therapeutic approach. This may be achieved by specifically targeting peripheral axons of nociceptors using therapeutic ointments and creams and/or applying drugs that reverse glycosylation onto DRGs using fluoroscopy-guided epidural and intrathecal injections in pain clinics.

### Glycosylation-Induced Alterations of Voltage-Dependent Properties of Ca_V_3.2 Currents

Biophysical properties of Ca_V_3.2 T-currents determine the threshold for current activation and inactivation which, in turn, determine the excitability of cells (Huguenard, 1996). It is generally acknowledged that enhanced cellular excitability of nociceptive DRG neurons directly translates into increased pain sensation *in vivo* (Campbell and Meyer, 2006; Calcutt, 2013). A question thus arises: Can glycosylation-induced alterations of the biophysical properties of T-channels alone alter the excitability of nociceptive neurons? Our data presented here (Figure 1) and in our earlier study (Orestes et al., 2013) indicate that glycosylated Ca_V_3.2 T-channels have significantly higher current densities. We continued here comparing the properties of voltage-dependent Ca_V_3.2 channel: deactivation, activation, steady-state inactivation, and recovery from inactivation in diabetic conditions using our standard protocols (Nelson and Todorovic, 2006; Nelson et al., 2007; Lee et al., 2009). Based on the results of our recordings with and without NEU treatment, we infer that glycosylation induces a significant hyperpolarizing shift in the steady-state activation and steady-state inactivation curves allowing a larger fraction of channels to open under a greater driving force. By contrast, glycosylation has little effect on current deactivation kinetics and recovery from inactivation. We speculate that negatively charged sialic acid residues from glycosylated groups may electrostatically affect external surface charges, which in turn may facilitate gating of the glycosylated Ca_V_3.2 channel. This could result in a hyperpolarizing shift of voltage-dependent activation and inactivation observed in our experiments. Facilitation of channel gating and increased current densities could certainly increase the excitability of nociceptive DRG neurons in diabetic animals. Hence, based on our study we propose that alterations in voltage gating and increased T-current densities due to glycosylation may contribute to hyperexcitability of DRG neurons in painful PDN. Although we are not aware of previously published reports on this topic, it is likely that other nociceptive ions channels in DRG similarly may be affected by glycosylation and could work in concert with Ca_V_3.2 to boost the excitability of nociceptors. For example, at least one study has shown that tetrodotoxin (TTX)-resistant I_Na+_ currents, which activate at a similar range potentials as T-currents, may also be modulated by glycosylation (Tyrrell et al., 2001). We hope that this issue can be directly addressed using mouse genetics in future current-clamp experiments using naïve DRG neurons and DRG neurons from the diabetic animals.

### Biochemical Evidence of Glycosylation in Native DRG Neurons

We have previously determined using recombinant Ca_V_3.2 channels that mutation of critical asparagine resides to glutamine such as N192Q and N272Q have unaltered membrane surface expression when compared with the WT Ca_V_3.2 in contrast to N1466Q which shows significantly decreased membrane expression in HEK293 cells grown in hyperglycemic conditions (Orestes et al., 2013). Furthermore, in the same study we demonstrated diminished current densities in N192Q mutants supporting the idea that different glycosylation sites on the Ca_V_3.2 protein may differently impact channel expression and channel activity. Because two of our point mutations are located in domain I of Ca_V_3.2, we generated N-terminus FLAG-tagged-Ca_V_3.2 (_6HIS/FLAG_Ca_V_3.2) to enable biochemical studies. We then used the FLAG-tag to immunoprecipitate the _6HIS/FLAG_Ca_V_3.2 channel from HEK293 cells grown in high glucose medium and treated with PNG to demonstrate using gel-shift analysis that the domain I of recombinant Ca_V_3.2 channel was glycosylated. Here we continued by using native DRG cells which allowed us to confirm our results in a more physiological cellular environment. Because in our previous studies the mobility of full-length recombinant Ca_V_3.2 was not noticeably affected by de-glycosylation (Orestes et al., 2013) we used here nickel-column chromatography to capture the N-terminal segment of the native DRG Ca_V_3.2 channels (Figure 10). Indeed our results demonstrate for the first time that native Ca_V_3.2 channels are glycosylated. If a higher degree of glycosylation occurs in STZ-induced diabetic DRG than DRG from healthy animals, we expected that enzymatic treatment may cause a greater decrease in the apparent MW of Ca_V_3.2-N-terminal fragments of Cav3.2 channels isolated from DRG tissue dissected from diabetic vs. healthy animals. In contrast, our gel-shift analysis suggests that N-terminal fragments of native Ca_V_3.2 channels in the DRG tissues are glycosylated to a similar degree in both healthy and diabetic animals.

However, we speculate that future extensive molecular studies and tandem mass spectrometry analysis of native tissues may be required to detect small differences (e.g., single amino acid) in the degree of Ca_V_3.2 channel glycosylation between diabetic vs. healthy DRGs. Such a premise is supported by our findings that i.pl. injections of NEU completely reversed hyperalgesia associated with Type 1 and Type 2 diabetes, while it had minimal effect on nociception in healthy animals (Orestes et al., 2013). This hypothesis is supported further by our new set of data presented herein showing that i.t. injections of NEU were more effective in increasing thermal PWLs in the diabetic than healthy rats.

## Conclusion

In conclusion, our current findings demonstrate that glycosylation-induced alterations in Ca_V_3.2 channels in sensory neurons *in vivo* may directly influence diabetic hyperalgesia, and that glycosylation inhibitors can be used to ameliorate painful symptoms in Type 1 diabetes. We expect that our studies may lead to a better understanding of the molecular underpinnings of painful PDN in an effort to facilitate the discovery of novel treatments for this intractable disease.

## Data Availability Statement

The raw data supporting the conclusions of this article will be made available by the authors, without undue reservation.

## Ethics Statement

The animal study was reviewed and approved by Animal Care and Use Committee of the University of Colorado Anschutz Medical Campus, as well as University of Virginia.

## Author Contributions

ST designed the electrophysiological and *in vivo* experiments. JE and PO performed electrophysiology experiments and analyses. SJ performed, analyzed *in vivo* experiments, and biochemical experiments. WM and PB designed biochemical experiments. WM performed and analyzed biochemical experiments. ST and SJ drafted the manuscript and all authors participated in revisions. ST, PB, and VJ-T were responsible for the overall direction of the project. All authors contributed to the article and approved the submitted version.

## Conflict of Interest

The authors declare that the research was conducted in the absence of any commercial or financial relationships that could be construed as a potential conflict of interest.
